# Across the space: applications of spatial transcriptomic technology in healthy and diseased muscle

**DOI:** 10.3389/fcell.2025.1656918

**Published:** 2025-10-21

**Authors:** Laura Virtanen, Chiara D’Ercole, Lorenzo Giordani

**Affiliations:** Sorbonne Université, INSERM UMRS974, Association Institut de Myologie, Centre de Recherche en Myologie, Paris, France

**Keywords:** skeletal muscle, spatial transcriptomics, muscle regeneration, muscle disorders, bioinformatics

## Abstract

In recent years, spatial transcriptomics (ST) has emerged as a groundbreaking technology with the potential to transform and accelerate our understanding of cellular crosstalk. While single-cell approaches have uncovered an unexpected level of cellular heterogeneity in both healthy and diseased tissues, they remain limited in their ability to capture cellular interactions in the native microenvironment. ST techniques bridge this gap by preserving anatomical information, enabling a direct investigation of spatially defined cellular interactions. This feature is particularly relevant in tissues such as skeletal muscle, where syncytial myofibers coexist with a heterogeneous set of interstitial cell populations. Spatial localization is a key factor during muscle regeneration, particularly as stem cell progression is driven by complex interactions between resident and recruited cell populations. Understanding these spatial dynamics is therefore critical to better characterize the fundamental mechanisms of muscle repair and identify aberrant signaling pathways of chronic injury or impaired regeneration. In this review, we will explore the various types of ST techniques, provide a brief summary of the available analytical tools, and highlight recent advancements in the skeletal muscle field enabled by the application of ST.

## 1 Introduction

Muscle regeneration relies on the coordinated interplay of diverse cell populations, which act together to maintain tissue homeostasis. Upon acute injury, tissue degeneration and necrosis are rapidly followed by the activation and expansion of muscle stem cells (MuSCs, also known as satellite cells). In parallel, different waves of recruited inflammatory cells, along with resident muscle cells, help dictate the timing and progression of the regenerative program. Eventually, a subset of activated MuSCs returns to quiescence, replenishing the stem cell pool and preserving long-term regenerative capacity (Dumont et al., 2015). This coordinated cellular activity ultimately leads to tissue remodeling and the restoration of muscle function (Mukund and Subramaniam, 2020).

Although regeneration in healthy muscle is highly efficient, this process can be dysregulated in pathological conditions such as muscular dystrophies and neurodegenerative disorders. For example, in Duchenne muscular dystrophy (DMD), a severe pediatric degenerative disorder, the widespread and asynchronous nature of injuries leads to chronic inflammation, contributing to regeneration impairment and ultimately resulting in fibrosis and fat infiltration (Dadgar et al., 2014).

In such complex pathological settings, where it is crucial to disentangle the cellular interactions driving disease progression, spatial transcriptomics (ST) offers a powerful advantage. Bulk and single-nucleus RNA sequencing (snRNA-seq) have been successfully employed to identify altered pathways and changes in cell populations in multiple contexts such as muscle injury (De Miche et al., 2020; Dell’Orso et al., 2019; Oprescu et al., 2020), denervation (Nicoletti et al., 2023; Lin et al., 2023), and in several muscle disorders (Chemello et al., 2020; Suárez-Calvet et al., 2023; Moncea et al., 2024). However, these approaches lack spatial information, limiting our understanding of cell-to-cell interactions and regional differences in cell signaling. ST overcomes this limitation by linking gene expression profiles to precise histological regions, making it an ideal tool for exploring the cellular and molecular landscape of muscle regeneration and degeneration.

In recent years, the number of publications using ST to study muscle physiology and pathophysiology has steadily increased (Moses et al., 2022). So far, ST approaches have been used to study muscle injury (Dadgar et al., 2014; Kan et al., 2024; Patsalos et al., 2024; McKellar et al., 2023; Stec et al., 2023; McKellar et al., 2021; Brorson et al., 2025; Larouche et al., 2023), denervation (D’Ercole et al., 2022), and neuromuscular disorders, such as Amyotrophic Lateral Sclerosis (ALS) (Ruggieri et al., 2025) and Duchenne Muscular Dystrophy (DMD) (Patsalos et al., 2024; Stec et al., 2023; Jeon et al., 2025; Heezen et al., 2023; Coulis et al., 2023; Young et al., 2022). Most of these studies have used multiomics approaches, integrating ST with reference snRNA-seq data to enhance spatial resolution. These pioneering efforts have validated ST as a valuable tool for studying cellular heterogeneity, cell–cell interactions, and the diffusion of signaling molecules between different areas. In this review, we will discuss the main ST techniques and key bioinformatic tools, highlight recent advances in muscle biology enabled by ST, and address current limitations as well as future perspectives.

## 2 Spatial transcriptomics technologies and bioinformatic tools

### 2.1 Current methodologies

**FIGURE 1:**
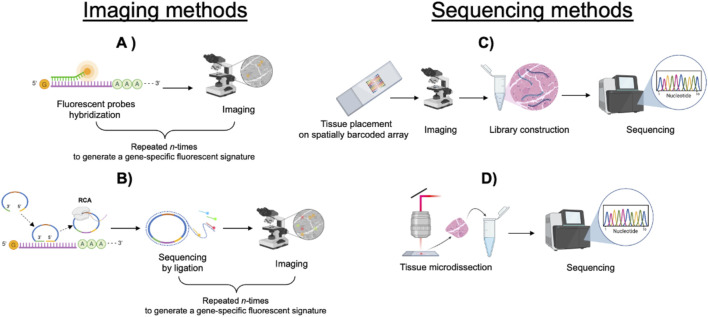
Diagram illustrating imaging and sequencing methods. **(A)** Fluorescent probes hybridize to specific sequences within the tissue and are imaged by microscopy. Repeated cycles of the procedure generate a gene-specific fluorescent signature. **(B)** Rolling circle amplification followed by sequencing by ligation. Repeated cycles of the procedure generate a gene-specific fluorescent signature. **(C)** Tissue placed on a barcoded array undergoes imaging, mRNA extraction, library construction, and sequencing. **(D)** Tissue microdissection followed by sequencing.

ST techniques can be broadly classified into two main categories: imaging-based methods and sequencing-based methods, the latter encompassing spatial array-based approaches and laser capture microdissection (LCM) (Figure 1).

*In situ* imaging-based techniques rely either on fluorescent *in situ* hybridization (ISH) or on *in situ* sequencing (ISS). Although the number of detectable transcripts may vary significantly depending on the different methods both techniques ultimately require a predefined set of targets, making them more suitable for hypothesis-driven rather than exploratory studies.

ISH-based techniques enable the visualization of RNA molecules directly within cells or tissues using fluorescently labelled probes complementary to target transcripts. Some of the most commonly used ISH techniques are: smFISH (Femino et al., 1998), RNA-scope (Wang et al., 2012), osmFISH (Codeluppi et al., 2018), seq-FISH (Lubeck et al., 2014), and MERFISH (Xia et al., 2019). SmFISH and RNA-scope are widely adopted for biological validation, as they rely on a small number of fluorescently labeled RNA probes (Femino et al., 1998). To address the initial limitations of smFISH various strategies and technical implementations have been developed expanding its applicability and sensitivity. In particular, RNAscope employs unique “double-Z” probes, in which two independent probe pairs (each shaped like a “Z”) must hybridize adjacently on the target RNA to initiate signal amplification. This dual-hybridization requirement enhances specificity and, combined with a series of amplification and hybridization steps, enables the detection of low-abundance RNA molecules (Wang et al., 2012). In osmFISH—an approach based on cyclic smFISH—unbarcoded, unamplified probes, labeled with a fluorescence tag, are hybridized to the tissue, imaged and subsequently removed by formamide melting. As each hybridization round is independent the number of detectable targets increases linearly with the number of cycles. However, a major limitation of the method is the progressive loss of RNA molecules with each round, resulting in an estimated ∼40% loss over 10 cycles (Codeluppi et al., 2018). To further increase multiplexing and minimize spectral overlap, seqFISH uses multiple rounds of hybridization in which the same probes are sequentially labeled with fluorophores of five different colors. This approach enables the generation of thousands of unique probe combinations (Lubeck et al., 2014). Building upon this concept, seqFISH+ expands the color palette to 60 “pseudocolor” channels through sequential hybridization, allowing the detection of up to 10,000 genes (Eng et al., 2019). To reduce the risk of errors due to the multiple rounds of amplification, MERFISH utilizes a combinatorial barcoding strategy to label transcripts before detection (Xia et al., 2019). Additionally, several of these methodologies have been adapted into automated commercial platforms, including MERSCOPE (MERFISH-based, Vizgen), Molecular Cartography (Resolve Biosciences), and CosMx (NanoString Technologies), which combines a MERSCOPE-like strategy with an optical signature approach comparable to Xenium (see below). The CosMx platform uses five gene-specific primary probes that hybridize to the target mRNA, followed by a fluorescently labeled secondary probe that binds to the primary probes. This process is iterated 16 times to generate a unique gene-specific signature, enabling the detection of up to 19,000 distinct transcripts (He et al., 2022).

ISS-methods are based instead on single-strand DNA probes complementary to a cDNA sequence, generated by reverse transcription of mRNA. Probes are hybridized on both sides of target sequence to form a rolling-circle product (RCP) which then undergoes sequencing (Ke et al., 2013). ISS- based technologies include: FISSEQ (Lee et al., 2014), HybISS (Gyllborg et al., 2020), ExSeq (Alon et al., 2021), and STARmap (Wang et al., 2018).

In FISSEQ the RNA is first reverse-transcribed to cDNA and then amplified and labeled with a fluorescent marker, followed by multiple rounds of sequencing *in situ* (Lee et al., 2014). To reduce the signal-to-noise ratio and improve the specificity HybISS-methodology takes advantage of lock-probes design by replacing random primers with specific sequences (Gyllborg et al., 2020). Instead, ExSeq relies on expansion microscopy to physically expand biological samples, to increase the precision of *in situ* RNA-seq while maintaining the overall structural integrity of tissues (Alon et al., 2021). STARmap method employs barcode-lock probes for direct targeting of over a thousand genes. The probes bind the cDNA to initiate a Rolling Circle Amplification (RCA); the RCA products are embedded in the hydrogel, allowing stable retention during multiple imaging cycles, amplification is then triggered only when both primers correctly hybridize to the target mRNA, ensuring high specificity. A major advantage of the STARmap is its applicability to thick sections (up to 150 µm), making it a powerful tool for exploring the three-dimensional organization of complex tissues (Wang et al., 2018).

In 2025, Maguire and colleagues introduced LIST-Lock-n-Roll (LIST-LnR) (Maguire et al., 2025). This *in situ* RNA detection method builds upon LISH technology (Ligation *in situ* Hybridization) - a technique developed to analyze RNA from formalin-fixed paraffin-embedded (FFPE) samples (Maguire et al., 2025). LIST-LnR relies on a specifically designed circularized probe system. Four unique readout probes, along with universal 5′ and 3′ bridge sequences, are appended to the respective ends of LISH probes. The construct is hybridized to the RNA target and amplified to create a rolling circle product (RCP). Each RCP is then identified based on the fluorescence emitted by the 5′fluorophores that are conjugated to the complementary readout probe sequences. Notably, this technique is compatible with both fresh frozen and formalin-fixed, paraffin-embedded specimens.

Among the commercially available platforms Xenium (10X Genomics) combines features from both ISS and ISH. Target sequences are initially subjected to a first round of hybridization with highly specific padlock probes, followed by a RCA amplification. Fluorescently labeled secondary probes then hybridize to the padlock probes, and an image is acquired. After imaging, the fluorescent probes are removed and replaced with new ones. The process is repeated on average eight times to generate a unique fluorescent signature that enables accurate gene identification (Janesick et al., 2023).

Xenium, MERSCOPE, and CosMx are the main image-based techniques used for profiling a variable number of genes at a sub-cellular resolution. While they share this core similarity, they differ in several key technical aspects, including gene panel design, sample types, and protein profiling capabilities. MERSCOPE allows for a fully customized gene panel (Moses et al., 2022) of up to 1,000 genes and thus can be applied to different species. In contrast, both Xenium and CosMx offer large mouse and human premade panels that can reach up to 5,000 genes and nearly 19,000 genes respectively. Both platforms also provide the possibility customize smaller probes panels both as adds-on or standalone (300 probes–CosMX and 480 - Xenium). Regarding protein profiling, MERSCOPE enables simultaneous RNA and protein detection on the same slide. CosMx performs this sequentially and offers custom panels specific to Human Immuno-Oncology or Mouse Neuroscience, with the option to add up to eight custom-conjugated antibodies to the existing protein panel (Lim et al., 2025). Xenium as well proposes a dedicated protein panel, which at present enables multiplexing of up to 27 proteins. Since these platforms continue to undergo rapid development, it is highly likely that their multiplexing capabilities will improve substantially in the near future.

Spatial array-based approaches rely on arrays of DNA-barcoded primers that carry spatial positional information. RNA molecules hybridize to these primers and are retrotranscribed, incorporating the spatial barcode into the resulting cDNA. As the precise location of each barcode on the slide is predetermined each transcript can be spatially mapped to its original position. Barcoded primers can be spotted on microarrays, attached on beads or nanoballs or directly to the tissue. In these systems, spatial resolution depends on the size of each spot and their relative distance within the array.

In their seminal 2016 publication Ståhl et al. (2016) coined the term ST–now broadly used to refer to the entire class of related technologies–to describe their method for detecting transcripts within their original tissue context (Ståhl et al., 2016). Their approach relied on a microarray of barcoded primers spotted directly on a slide with each spot measuring 100 µm in diameter. Based on this technology a commercial version was released in 2019 (Visium V1–10x Genomics) featuring approximately 5,000 spots (55 µm-diameter) per capture area, each containing around five million barcoded oligonucleotides. Tissue sections must be carefully placed over the barcoded capture area and, after permeabilization, mRNA molecules hybridize to spatially barcoded primers, allowing reverse transcription into cDNA. Once the tissue is enzymatically removed, the synthesized cDNAs hybridize with probes on the slide for further processing. Prior to this step, either immunofluorescence or hematoxylin and eosin (H&E) staining is performed to enable accurate alignment of histological features with the corresponding sequencing data. Visium V1 platform relies on a 3′poly(A) capture-based chemistry. Accurate placement of tissue samples on the capture area remains a significant technical challenge for this initial version. To address this issue, later versions of the Visium workflow incorporated the CytAssist instrument to ensure accurate transfer of tissue sections onto the slide, together with the probe-based chemistry introduced in Visium V2. Building on Visium V1 technology McKellar and colleagues developed STRS (spatial total RNA-sequencing) which by introducing a step to add a poly(A) tail to the 3′ end of all RNAs enables the additional detection of non-coding RNAs and viral RNAs (McKellar et al., 2023). Recently, 10x Genomics released a high-resolution version of the Visium platform (Visium HD), available in two formats: one using probe-based chemistry and another using 3′poly(A) capturere, both employing a high-density array that achieves ∼2 µm resolution.

In the Slide-seq approach, a tissue section is placed onto a surface covered with DNA-barcoded beads with known spatial positions (Maguire et al., 2025). The mRNA released from the tissue is captured by the beads and used to generate 3′-end, barcoded RNA-seq libraries; the purified cDNA is then subjected to next-generation sequencing (NGS). Slide-seq provides a spatial resolution of 10 μm and is commercially available under the name Curio Seeker (Curio Bioscience) (Rodriques et al., 2019). In 2021, Curio Bioscience released an improved version called Slide-seqV2 that achieves a ∼10-fold increase in RNA capture efficiency compared to the original version (Stickels et al., 2021).

Similarly to Slide-seq, HDST achieves subcellular resolution (∼2 µm) by randomly distributing uniquely barcoded beads into a densely packed hexagonal array of 2 µm wells. The precise location of each bead is then decoded through sequential hybridization and imaging of fluorescently labeled oligonucleotides. This process assigns each bead a unique spatial color address, which, enables accurate spatial mapping of captured mRNAs (Vickovic et al., 2019).

DBiT-seq introduces spatial barcodes into tissue sections via orthogonally applied microfluidic channels. This process results in the *in situ* labeling of mRNA molecules, which are reverse transcribed into barcoded cDNAs. Additionally, this method supports multi-omic profiling, enabling concurrent analysis of transcriptomic and proteomic data by incorporating antibody-derived DNA tags (ADTs) (Liu et al., 2020).

Seq-Scope enables high-efficiency mRNA capture through a PCR-based *in situ* method. The RNA capturing array is generated by solid-phase amplification of random barcode molecules using an Illumina sequencing platform. This process yields a center-to-center resolution of approximately 0.5–0.8 μm. Such ultra-high spatial resolution enables transcriptomic profiling at tissue, cellular, and subcellular levels (Cho et al., 2021).

In sci-Space, spatial barcoding is achieved by transferring spatially arrayed hashed oligonucleotides onto a tissue section to label nuclei locations (Srivatsan et al., 2020). A regular grid of hashed oligos is spotted onto agarose-coated slides, which are then physically juxtaposed to the tissue to enable transfer. During this process, the tissue is imaged and subsequently dissociated to isolate single nuclei, which are then subjected to sci-RNA-seq (single-cell RNA sequencing with combinatorial indexing). Upon sequencing, the approximate location of each nucleus can be inferred based on its associated hashed oligos. Unlike classical array-based approaches, where the spatially barcoded spot is sequenced, sci-Space sequences the extracted, labeled nucleus. In this sense, sci-Space could be considered a spatially informed snRNA-seq method. While this approach does not provide precise transcript localization, it offers single-cell resolution across large areas, making it well suited for mapping broad tissue regions (Srivatsan et al., 2020).

Stereo-seq relies on a DNA nanoball (DNB) patterned array; this approach, enables a resolution of 0.22 μm–with approximately 400 spots for tissue RNA capture per 100 μm^2^. DNB templates containing random barcodes are deposited on the patterned array, incubated with primers, and sequenced to obtain the coordinate identity of every experiment. Next, UMIs and polyT oligos for RNA capture are added to the DNB. Tissue sections are then placed on the chip, and after fixation and reverse transcription, the barcoded cDNA is sequenced. Stereo-seq provides higher spatial resolution combined with larger capture areas (up to 13.2 × 13.2 cm), making it suitable for profiling tissues of various sizes, including whole mouse embryos (Chen et al., 2022).

GeoMx Digital Spatial Profiling (NanoString Technologies) is able to quantify the abundance of protein or RNA by counting unique indexing oligonucleotides assigned to each target in a specific region of interest (ROI). Gene-specific probes (or primary antibody) are covalently attached to the indexing oligonucleotides and first hybridized to the targets within the tissue (Merritt et al., 2020). These probes are linked to unique barcodes through UV-cleavable linkers. The tissue slide is then stained with fluorescently labeled imaging probes to visualize specific cell types. Imaging data are then used to guide ROI selection. UV light is applied to these selected ROIs to release the barcodes, which are then collected for library preparation and sequencing.

To analyze gene expression in specific tissue regions, LCM-based ST relies on the precise laser microdissection of regions of interest (ROI) followed by high-throughput sequencing. Several approaches have been developed over the years including LCM-seq (Nichterwitz et al., 2016), TIVA (Lovatt et al., 2014), Tomo-seq (Junker et al., 2014), Geo-seq (Chen et al., 2017), NICHE-seq (Medaglia et al., 2017), and ProximID (Boisset et al., 2018). LCM-seq (Nichterwitz et al., 2016), combines laser-capture microdissection with RNA sequencing, enabling the analysis of single cells or small tissue regions with high precision. TIVA (Lovatt et al., 2014) (Transcriptome *In Vivo* Analysis) employs a photoactivatable probe to isolate mRNA directly from living cells, maintaining their native environment. Tomo-seq (Junker et al., 2014) provides spatial maps of gene expression by sequencing consecutive tissue sections along a selected axis. Similarly, Geo-seq (Chen et al., 2017) combines LCM and scRNA-seq to reconstruct transcriptomic landscapes while retaining spatial information. To focus on immune cells, NICHE-seq (Medaglia et al., 2017) integrates photoactivation with single-cell sequencing to profile specific populations within defined tissue microenvironments. Finally, ProximID (Boisset et al., 2018) identifies the transcriptomes of physically interacting cells, offering valuable insights into cell–cell communication at the molecular level. All these methods require frozen tissue sections except for TIVA, which is able to capture mRNA from live cells using a biotin-tag. Microdissection-based approaches enable precise analysis of microanatomical structures and gene expression by directly selecting specific regions (typically 60–700 μm in diameter); however, they are often limited by the quantity and quality of RNA, as molecules may be compromised during dissection and processing (Du et al., 2023).

The selection of the most appropriate methodology depends on multiple factors and requires a careful evaluation of the advantages and limitations of each technology. In particular, three key factors should be taken into account: the number of required detectable targets, detection efficiency and spatial resolution (for a summary of the major commercially available platforms see Table 1). The first step is to clearly define the scientific question—specifically, whether the study requires comprehensive whole-transcriptome coverage or focuses on a targeted subset of transcripts. This decision determines whether a sequencing-based or imaging-based approach is more suitable. 1maging-based methods typically offer higher spatial resolution and sensitivity, making them ideal for studies involving well-defined transcript panels. However, their use is largely limited to human and mouse samples due to probe availability. In contrast, sequencing-based methods generally provide whole-transcriptome coverage but at the cost of lower resolution and sensitivity (Valihrach et al., 2024). The vast majority of ST studies conducted on muscle tissue have so far relied on array-based technologies (Kan et al., 2024; Patsalos et al., 2024; McKellar et al., 2023; Stec et al., 2023; McKellar et al., 2021; D’Ercole et al., 2022; Ruggieri et al., 2025; Jeon et al., 2025; Heezen et al., 2023; Coulis et al., 2023; Young et al., 2022; Walter et al., 2024). This trend can be attributed to the relatively recent adoption of spatial approaches in this specific tissue context, with most exploratory studies dating back to late 2020. These early investigations have primarily focused on broad transcriptional mapping, for which array-based platforms provided a practical and accessible entry point. As previously discussed, the choice of ST technology is tightly linked to the specific scientific question being addressed. For this reason, while array-based methods currently dominate the field, imaging-based approaches should not be overlooked for future studies - especially as research increasingly shifts toward more targeted and high-resolution analyses. Moreover, given the constantly increasing number of detectable probes, it is foreseeable that probe-based platforms will eventually replace spot-array systems—indeed, full-genome scale panels are already available on platforms such as CosMX. However, in the context of skeletal muscle research, the relative novelty of these technologies and the lack of large-scale, comparable datasets make it difficult to directly evaluate the sensitivity and performance of high-resolution array systems (e.g., Visium HD) against probe-based approaches using large or full-genome panels. Future benchmarking studies across different tissues and conditions will therefore be essential to determine the most suitable strategies for specific research applications.

**TABLE 1:** Commercially available spatial transcriptomic platforms.

Methods	Platforms	Provider	Sample type	Suitable Species	Instrument required	Spatial resolution	Gene coverage
Spatial array-based	Visium	10X Genomics	V1: FF V2: FF, FFPE, FxF	V1: Any species V2: Mouse and Human	V1: NA V2: Visium CytAssist	55 μm	Whole transcriptome
	Visium HD	10X Genomics	Probes: FF, FFPE, FxF 3’: FF	Probes: Mouse and Human 3’: Any species	Visium CytAssist	2 μm	Whole transcriptome
	GeoMx DSP	NanoString Technologies	FF, FFPE	Mouse and Human	GeoMx DSP	10 μm	Whole transcriptome
	Curio Seeker	Curio Biosciences	FF	Any species	NA	10 μm	Whole transcriptome
	Stero-seq/STOmics	BGI Genomics	FF, FFPE	Any species	STOmics chip reader	0.22 µm	Whole transcriptome
Imaging- based	Molecular Cartography	Resolve Bioscience	FF, FFPE	Any species	Molecular Cartography	0.3 µm	100 genes
	Visium Xenium	10X Genomics	FF, FFPE	Mouse and Human	Xenium Analyzer	Subcellular	Up to 5,000 genes (Customizable additional genes)
	MERSCOPE	Vizgen	FF, FFPE	Any species	MERSCOPE Ultra™	Subcellular	Up to 1,000 genes
	CosMx SMI	NanoString Technologies	FF, FFPE	Mouse and Human	CosMx Spatial Molecular Imager	Subcellular	Up to 19,000 genes (Customizable additional genes)

FF, fresh frozen; FFPE, formalin-fixed paraffin-embedded; FxF, fixed frozen; NA, not available.

### 2.2 Bioinformatics analysis and tools

ST data analysis is a multi-step process aimed at extracting biologically relevant information by leveraging both spatial coordinates and gene expression data. The analysis begins with data preprocessing, which generates a gene expression matrix along with the corresponding spatial coordinates. This step depends highly on the underlying technology. Each preprocessing pipeline is tailored to the specific technical requirements in terms of input for the employed method, yet they all ultimately generate a gene count matrix. This matrix represents gene expression levels within individual spatial units, which correspond to single RNA molecules in imaging-based techniques (or to cells if combined with segmentation) and spots in sequencing-based approaches. Tools such as Starfish (Axelrod et al., 2021) provide scalable pipelines for processing image-based transcriptomics, allowing localization and quantification of RNA transcripts within image data (Axelrod et al., 2021; Perkel, 2019). Preprocessing for sequencing-based ST includes aligning sequencing reads, processing tissue images, and matching spatial barcodes to produce spatial gene expression matrices. Subsequent analyses applied to the matrix are largely shared across different ST platforms (Valihrach et al., 2024). Commercial platforms typically provide dedicated proprietary software and pipelines for data visualization and analysis. For instance, Visium workflow relies on Space Ranger for read alignment and quantification and Loupe Browser for interactive data visualization. Stereo-seq employs tools like ImageStudio for quality assessment and SAW to generate matrices compatible with downstream analysis. Once the preprocessing is complete, data undergoes normalization, dimensional reduction, and clustering. To streamline this process, several computational frameworks such as Seurat (Hao et al., 2021; Stuart et al., 2019), Squidpy (Palla et al., 2022), STUtility (Bergenstråhle et al., 2020) have been developed. These frameworks also provide comprehensive analysis and visualization tools. Regarding dimensionality reduction, the most commonly adopted methods, such as PCA (Principal Component Analysis) (Jolliffe and Cadima, 2016), t-SNE (t-distributed Stochastic Neighbor Embedding) (Van Der Maaten and Hinton, 2008), and UMAP (Uniform Manifold Approximation and Projection) (McInnes et al., 2018), are directly borrowed from scRNA-seq analysis workflows.

One of the key advantages of ST is the possibility to identify spatially distinct gene expression regions which often overlap with histological and functional tissue domains. While standard clustering strategies [k-means (Ikotun et al., 2023), Louvain (Blondel et al., 2008)] remain widely used, several new methods have recently been developed to improve clustering accuracy and a better identification of gene-coherent spatial domains. These include tools such as BayesSpace (Zhao et al., 2021) and SC-MEB (Yang et al., 2022), both based on Bayesian statistics, and StLearn (Pham et al., 2023), which offers a suite of algorithms to construct pseudo-time-space trajectories, expand ST coverage, and analyze cell-cell interactions. Additionally, several tools rely on deep learning strategies including DeepST (Xu et al., 2022), SpaGCN (Hu et al., 2021), STAGATE (Dong and Zhang, 2022) and SiGRA (Tang et al., 2023a) which can process multichannel images as input.

At present, several ST techniques can achieve cellular and subcellular-level resolution, and a wide range of bioinformatic tools have been developed to annotate cell types and investigate their interactions. For *in situ* imaging approaches, an essential step is image segmentation, which enables the generation of single-cell level information. Tools such DeepCell (Van Valen et al., 2016), CellPose (Stringer et al., 2021) or Stardist (Weigert and Schmidt, 2022) are commonly used to identify nuclei or cells in high resolution tissue images (Kleino et al., 2022). However, in tissues with a more complex organization, such as skeletal muscle, additional segmentation strategies are required to accurately capture structural and functional units. Due to its multinucleated nature, skeletal muscle requires an additional segmentation step to identify individual fibers to fully analyze tissue architecture and regeneration. Historically, manual segmentation has been used to delineate and measure single fibers and their characteristics; however, this approach is time-consuming and lacks scalability. In recent years, various bioinformatic tools have become available enabling automated segmentation of muscle fibers. In particular general-purpose segmentation tools such as Cellpose, which relies on a deep learning-based segmentation algorithm, has been successfully applied to murine skeletal muscle, accurately segmenting thousands of myofibers in fixed tissue (Waisman et al., 2021). Other machine learning-based tools, such as Ilastik (Berg et al., 2019), can also be used in principle to reliably identify muscle fibers. Moreover, several dedicated standalone tools [Myotally (Both et al., 2025) or MyoV (Gu et al., 2024)] or Fiji plugins are now available MuscleJ (Mayeuf-Louchart et al., 2018), Open-CSAM (Desgeorges et al., 2019), MyoSAT (Stevens et al., 2020), Myosoft (Encarnacion-Rivera et al., 2020) and MyoView (Rahmati and Rashno, 2021). While these tools are primarily designed to measure and analyze fiber type and morphological characteristics, their segmented labels can be imported into computational frameworks such as SpatialData (Marconato et al., 2025), to be combined with high-resolution ST data and generate single-cell–level datasets.

For techniques with lower resolution, such as Visium, a deconvolution step is often required. Typically using a scRNA-seq reference to estimate the relative cell type composition of each spot. Tools such as SPOTlight (Elosua-Bayes et al., 2021), SpatialDWLS (Dong and Yuan, 2021), Cell2location (Kleshchevnikov et al., 2022) CellTrek (Wei et al., 2022), RCTD (Cable et al., 2022), DSTG (Song and Su, 2021), GraphST (Long et al., 2023), Tangram (Biancalani et al., 2021), and STRIDE (Sun et al., 2022) utilize diverse computational strategies to reconstruct the spatial distribution of cell types with high resolution. Another strength of ST is its ability to detect active signaling and infer intercellular communication reliably. Since signaling efficiency depends on the distance between cells, linking gene expression to spatial coordinates allows for more precise modeling of these interactions. This represents a marked difference from scRNAseq-based-inference, where the complete lack of spatial context can lead to false-positive predictions. Several tools such as Cellchat v2 (Jin et al., 2024), CNG (Yuan and Bar-Joseph, 2020), SpaOTsc (Cang and Nie, 2020), MISTy (Tanevski et al., 2022), and spaCI (Tang et al., 2023b) are currently available to model ligand-receptor interactions within tissue microenvironments.

Lastly, trajectory inference methods can also be used to reconstruct the dynamic processes of cell development and differentiation over spatial gradients. StLearn (Pham et al., 2023) offers a Pseudo-Spatial-Time (PST) model, while SPATA (Kueckelhaus et al., 2024) uses monocle3-based analysis to infer transcriptional changes along spatial trajectories, enabling the study of temporal and developmental tissue organization.

**FIGURE 2:**
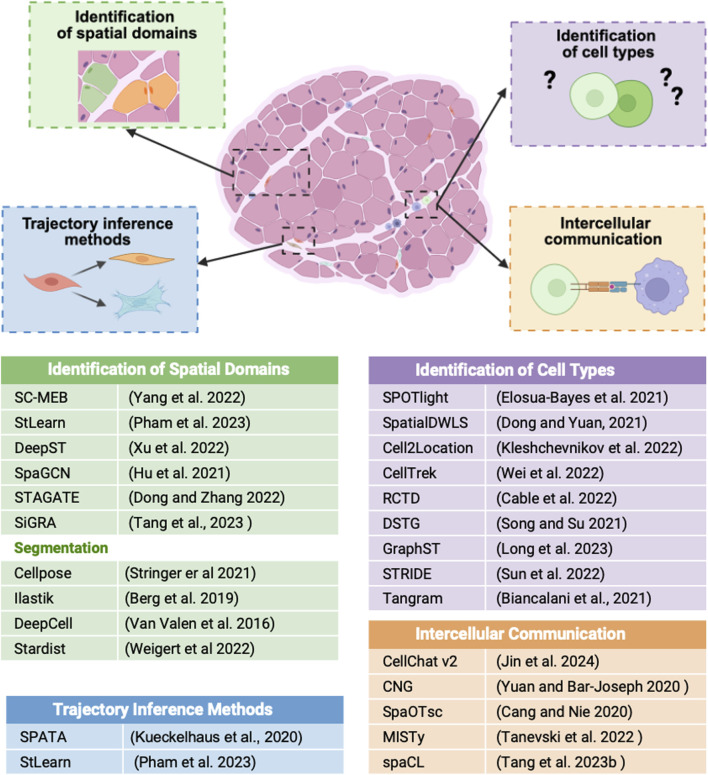
Schematic illustration showing how ST data can be used to identify spatial domains, cell types, cellular trajectories, and intercellular communication within tissue sections. Each process is linked to the bioinformatic tools listed in the table at the bottom.

Together, these tools and computational strategies provide a robust framework for interpreting ST data and gaining insights into tissue biology (Figure 2).

### 2.3 Omics integration

The integration of multi-omics, such as genomics, epigenomics, transcriptomics, proteomics, or metabolomics, can reveal how biological processes are coordinated at multiple molecular levels.

Spatial multi-omic profiling of tissue samples can be achieved either by applying spatial mono-omic assays separately on serial sections, or by employing integrated strategies that capture multiple omic layers within the same tissue section. In the sequential approach, fresh-frozen (FF) or formalin-fixed paraffin-embedded (FFPE) tissue sections are analyzed using different spatial methods, often combined with morphological staining and annotations, followed by computational integration of the datasets. A key challenge with this approach is the accurate alignment of serial sections, as tissue stretching or deformation during sectioning can hinder precise image registration (Vandereyken et al., 2023).

Several studies have demonstrated the value of these combined strategies. For example, in 2021, ST was combined with spatial proteomics to analyze high-grade muscle-invasive bladder cancer (MIBC) samples (Gouin et al., 2021). In that study, ST data served as the primary analytical focus, while spatial proteomic data was used to validate the transcriptomic findings. Similarly, integration of spatial metabolomics and ST has also been performed, where the metabolomic data formed the core of the analysis and was supported by ST validation. This study employed a novel method called Spatial Single-Nuclear Metabolomics (SEAM) (Yuan et al., 2021) to map metabolite distributions in human fibrotic liver tissue.

In parallel, additional multi-omic strategies are being developed to enable simultaneous spatial profiling of multiple molecular modalities within the same tissue section. For instance, spatial ATAC&RNA-seq and spatial CUT&Tag-RNA-seq (Zhang et al., 2023) allow gene expression and chromatin features to be studied together in the same tissue slices. These approaches combine DBiT-seq (Liu et al., 2020) based methods with spatial-ATACseq (Deng et al., 2022a) or spatial-CUT&Tag (Deng et al., 2022b) to map open DNA or histone marks along with mRNA. Advanced fluorescence *in situ* hybridization (FISH)-based methods, including MERFISH (Xia et al., 2019) and seqFISH+ (Eng et al., 2019) use predefined optical barcodes and complex probe designs to visualize thousands of RNA transcripts and genomic loci in single cells, with optional limited protein detection *via* fluorescent or DNA-conjugated antibodies. Array-based technologies (Ståhl et al., 2016) such as Visium can be paired with hematoxylin and eosin (H&E) staining or limited antibody-based protein profiling.

Extended platforms like SM-Omics (Vickovic et al., 2022) further allow co-detection of multiple proteins *via* antibody-derived tag (ADT)-conjugated antibodies. Finally, NanoString’s GeoMx (Merritt et al., 2020) enables quantification of RNAs and proteins within selected regions of interest (ROIs). This is achieved by UV-photocleavable linkers that release uniquely barcoded oligonucleotides from antibodies or probes upon UV illumination. The released barcodes are then collected and sequenced, allowing spatial mapping on the original tissue.

## 3 Applications of ST in skeletal muscle research

In the field of skeletal muscle, ST has found multiple applications in healthy, regenerating, and diseased muscle (for a summary of cited studies see Table 2). This chapter highlights the key findings enabled by ST across these biological settings.

**TABLE 2:** Applications of spatial transcriptomic technology in healthy and diseased muscle.

Author	Muscles	Injury	Specie/Strain	Age	Technique
Brorson et al (2025)	VL	Ctrl, 2, 8, 30 dpi (electric)	Human	55–80 yr	Visium 10x Genomics
Jeon et al (2025)	Quadriceps, abdominal		Healthy, DMD	17 yr	Visium 10X Genomics MERFISH
Martínez et al (2024)	TA		Polr2b–DCM mice Tg (Pax7-EGFP) 15Tajb mice	4–6 mo	Tomo-seq, CODEX
Ruggieri et al (2025)	TA		C57BL/6J SOD1^G93A^ mice	2.5 mo 2.5–4.5 mo	Visium 10X Genomics
Umek et al. (2025)	VL		Healthy, sarcopenia	85 yr, 19 yr	Xenium 10X Genomics
Kan et al (2024)	TA	Ctrl 1, 3, 7 dpi (NTX)	Nse-Bmp4 transgenic mice		Visium 10x Genomics
Mcleod et al (2024)	VL		Human	<50 yr	MERFISH
Patsalos et al (2024)	TA GA	4 dpi (CTX)	C57BL/6J DBA/2J-*mdx* mice	2 mo	Visium 10X Genomics
Walter et al (2024)	TA	5 dpi (NTX)	BL/6J mice	4–7 mo, 26 mo	Curio Seeker (Slide-seq) Curio Bioscience
Coulis et al (2023)	GA, Plantaris		DBA/2J-*mdx* mice	<2 mo	Visium 10X Genomics
Heezen et al (2023)	Quadriceps		C57BL/6J-*mdx* DBA/2J, DBA/2J-*mdx* mice	2.5 mo	Visium 10X Genomics
McKellar et al (2023)	TA	Ctrl 2, 5, 7 dpi (NTX)	C57BL/6J mice	6 mo	Visium 10X Genomics STRS
Larouche et al (2023)	TA	7, 14 dpi (VLM)	C57BL/6J mice		Visium 10X Genomics
Stec et al (2023)	GA	1, 3, 5 dpi (CTX)	C57BL/6J, DBA/2J *mdx*, DBA/2J-*mdx* mice	< 2 mo	Visium 10X Genomics
D’Ercole et al (2022)	TA	Ctrl 3, 30 dpi (denervation)	C57BL/6J mice	4–7 mo	Visium 10X Genomics
Young et al (2022)	GA		DBA/2J-*mdx* mice	2 mo	Visium 10X Genomics
McKellar et al (2021)	TA	2, 5, 7 dpi (NTX)	C57BL/6J mice	5 mo	Visium 10X Genomics
Dadgar et al. (2014)	GA	Multiple NTX	C57BL/6J mice Human DMD	4–8 mo 3 yr	LCM

VL, vastus lateralis; TA, tibialis anterior; EDL, extensor digitorum longus; GA, gastrocnemius; CTX, cardiotoxin; NTX, notexin; Dpi, days post injury; IF, immunofluorescent staining; H and E, hematoxylin and eosin staining; Mo, month; Yr, year; Ctrl, control; DMD, duchenne muscular dystrophy.

### 3.1 Healthy and regenerating muscle

The initial applications of ST in skeletal muscle date back to 2021 when McKellar and colleagues, Using the Visium platform, explored cell–cell interactions during acute muscle regeneration (McKellar et al., 2021). To enrich the relatively rare intermediate myogenic cell populations in healthy muscle, the authors collected TA muscles from 5-month-old C57BL/6J mice subjected to myotoxin-induced injury at 2, 5, and 7 days post-injury (dpi). The ST data were integrated with 23 newly generated and 88 publicly available single-cell and single-nucleus RNA-seq datasets to perform deconvolution of cell subtypes. Through snRNA-seq–based deconvolution, the authors identified and distinguished quiescent muscle stem cells (MuSCs), activated MuSCs, committed myoblasts, fusing myocytes, and mature myonuclei. Additional cell types, including neural cells, tenocytes, smooth muscle cells, and several subpopulations of fibro-adipogenic progenitors (FAPs), endothelial cells, and immune cells, were also detected.

At 2 dpi, the injury site showed increased expression of *Myod1*, indicative of MuSC activation, and a lack of mature myosin gene *Myh1*. By five and 7 dpi, the expression of cell cycle inhibitors such as *Cdkn1c*, the myogenic commitment marker *Myog*, and fusogenic genes including *Mymk* and *Mymx* became enriched. Simultaneously, pro-remodeling FAPs increased, while patrolling monocytes declined, indicating the progression of the regenerative response.

According to a cell–cell interaction analysis, FAPs exhibited the highest predicted levels of interaction with myogenic cells through secreted signaling. These interactions decreased as myogenic cells progressed towards more differentiated myogenic stages. Notably, pro-remodeling FAPs showed high expression of a secreted growth factor *Midkine* (*Mdk*), while myogenic cells, particularly quiescent MuSCs, expressed its receptor genes *Ncl*, *Sdc4*, *and Lrp1*. Moreover, *Mdk* was spatially co-expressed with *Ncl* and *Lrp1*, suggesting a coordinated paracrine signaling mechanism. *Mdk* signaling has previously been implicated in limb regeneration (Qin et al., 2021) and the regulation of stem cell proliferation (Xu et al., 2014). In conclusion, these findings demonstrate how ST, integrated with single-cell data, can identify different cell subtypes and resolve dynamic cell interactions during muscle regeneration.

In 2023, the same group introduced Spatial Total RNA Sequencing (STRS), a method designed to capture coding, non-coding, and viral RNAs. With this approach, they identified spatially defined distributions of non-coding RNAs during muscle regeneration (McKellar et al., 2023). They collected TA muscles from 6-month-old C57BL/6J mice subjected to myotoxin-induced injury at 2, 5, and 7 dpi in addition to an uninjured control. Gene expression analysis revealed that the long non-coding RNA *Meg3* was expressed at the injury site at 5 dpi, which is a critical time point for myoblast differentiation and myocyte fusion (McKellar et al., 2023). This aligns with prior *in vitro* findings demonstrating the role of *Meg3* in myoblast differentiation (Dill et al., 2020). Additionally, consistent with earlier studies (Liu et al., 2012) *miR-1a-3p* was expressed at all time points, while *miR-206-3p* showed high expression only at five dpi in the injury site. These results demonstrate that STRS can capture RNAs that are missed by conventional workflows, including non-coding RNAs, newly transcribed RNAs and viral RNAs.

Additional insights into the mechanisms underlying muscle regeneration were provided by Young et al. (2022). By reanalyzing ST data from McKellar on myotoxin-induced muscle injury, the authors identified a senescence-associated gene signature (defined by genes such as *Trp53*, *Cdkn1a*, and *Cdkn1c*) enriched within degenerating muscle regions. They then extended their investigation to a model of chronic injury, the D2-mdx mouse model, using the Visium platform to assess the spatial distribution of this senescence signature. Their analysis revealed that the signature was enriched in areas undergoing active muscle repair. Importantly, pharmacological depletion of senescent cells using senolytic agents impaired muscle growth following injury, suggesting that transient senescent cell accumulation is a necessary component of effective regeneration.

Our group, in collaboration with the group of Luca Madaro at Sapienza University, focused on muscle denervation and leveraged the Visium platform to investigate its effect at the whole tissue level. By integrating ST with immunofluorescence, we demonstrated a clear correspondence between unbiased ST regions and anatomical-functional domains within skeletal muscle (D’Ercole et al., 2022). We validated these functional regions using immunofluorescence and histochemical assays, confirming the localization of various anatomical structures, including different fiber types, the epimysium, blood vessels, nerves, and the neuromuscular junction (NMJ). Furthermore, we were able to clearly distinguish between different types of myofibers using specific markers like *Myh1*, *Myh2*, and *Myh4* and confirmed the spatial gene distribution with immunofluorescence staining. Building on this spatial framework, we investigated the molecular response of these regions to reversible denervation. Using a spatiotemporal approach, we tracked transcriptomic changes within each domain of the TA muscle 3 and 30 days following sciatic nerve compression. Our analysis revealed a marked dysregulation of the polyamine pathway specifically in the glycolytic region 3 days post-denervation, resulting in the accumulation of putrescine. Moreover, changes in the expression patterns of *Amd1*, *Amd2*, and *Smox*—genes involved in the polyamine pathway—correlated directly with muscle atrophy. Consistently, increased expression of the pro-atrophic markers *Atrogin-1* (*Fbxo32*) and *Murf1* (*Trim63*) was detected in type IIB fibers of the TA muscle after denervation. *In vitro* experiments also showed that putrescine accumulation can induce *Trim63* expression, while genetic inactivation of the polyamine pathway in *Drosophila* reduced muscle function. The findings of this work highlight how the atrophic signaling pathway and polyamine metabolism are spatially organized and nerve-dependent in glycolytic fibers (D’Ercole et al., 2022).

Other studies have examined muscle regeneration during aging or in response to volumetric muscle loss. For instance, Walter and colleagues generated a large-scale single-cell aging atlas of regenerating muscle and identified gene signatures specific to young aged BL6 mouse muscle-resident cells (Walter et al., 2024). Using the CurioSeeker platform, they explored the effect of aging on the accumulation of senescent-like MuSCs and progenitor cells in TA muscle in 5 dpi regions of young and geriatric samples. Cell type deconvolution revealed a higher fraction of fusing myocytes and FAPs in the young injured zone, and a greater abundance of monocytes/macrophages, T cells, and myonuclei in the geriatric injured zone. Furthermore, by applying a senescence-associated gene signature, they showed that MuSCs and progenitors exhibited an elevated senescence score in the geriatric injured area, whereas non-MuSCs showed higher scores in the non-injured zone.

Extending the use of ST to a different pathological setting, Larouche and colleagues utilized ST to investigate the tissue response to volumetric muscle loss (VML) (Larouche et al., 2023). VML was performed on the TA of BL6 mice using punch biopsies, and tissue was collected at 7 dpi. To decipher the mechanisms underlying regeneration, the tissue was divided into zones of complete muscle loss (defect zone) and remaining intact muscle (intact zone). By analyzing the transcript distribution across the intact and defect regions, they observed that macrophages and mesenchymal-derived cells (MDCs) preferentially infiltrated the damaged area, while MuSCs were retained in the intact muscle. The authors proposed that the absence of MuSCs from the injury site was caused by a hostile environment characterized by biophysical factors, such as matrix alterations, and the presence of pro-fibrotic signals. This hypothesis is supported by the colocalization of pro-fibrotic signaling molecules, such as TGF-β, with macrophages and MDCs. Furthermore, pharmacological blockade of this cellular crosstalk, *via* TGF-β receptor 2 (TGFBR2) inhibition, was shown to promote tissue regeneration and attenuate both inflammatory and fibrotic processes.

ST has also been applied to investigate muscle adaptation and response to physiological stimuli. McLeod and collaborators investigated non-coding RNA (ncRNA) expression during human skeletal muscle hypertrophic remodeling (Mcleod et al., 2024). In their work McLeod and colleagues analyzed 288 transcriptome-wide profiles and found 110 ncRNAs linked to muscle growth *in vivo.* Subsequently, they used MERSCOPE to map probes targeting both well-established muscle cell marker genes and ncRNAs. This approach enabled the identification of novel ncRNAs and chart their localization in the hypertrophic muscle. For example, the expression of *MYREM* (MYBPC2 cis-regulating lncRNA enhancer of myogenesis) was found beneath the basal lamina, but not in the nuclei of satellite or endothelial cells, suggesting that *MYREM* could be a novel marker of mature myonuclei, particularly in type II fibers. This is further supported by recent data showing that *MYREM* upregulates *MYBPC2*, which encodes a type II isoform of myosin-binding protein C in skeletal muscle. In contrast, *MEG3* was co-localized with satellite cells, aligning with other studies demonstrating its role in myoblast differentiation (McKellar et al., 2023; Dill et al., 2020). Additionally, *CARMN* (cardiac mesoderm enhancer-associated non-coding RNA) was associated with endothelial cells and pericytes, suggesting that *CARMN* may modulate pericyte function during skeletal muscle growth. Collectively, these observations reveal distinct ncRNA localization patterns in hypertrophic muscle.

Martínez Mir et al. (2024) studied fiber-type composition by combining RNA tomography (Tomo-seq) with mass spectrometry imaging (MSI), enabling simultaneous analysis of the ST, metabolomic, and lipidomic organization of mouse TA muscle. Notably, they examined both transverse and longitudinal orientations of the muscle to capture regional variation. To assess transcript distribution, Tomo-seq was applied to TA muscles from 4- to 6- month-old mice, along the proximal–distal axis. Unsupervised clustering identified three distinct molecularly defined regions: proximal, central, and distal. Proximal–distal regions were enriched for genes associated with glycolytic fiber types (*Myh4*, *Myl1)*, the myotendinous junction (*Prg4*, *Thbs4*, *Itgb1*, *Col1a2*), and glycolytic metabolism (*Pygm*). In contrast, the central region was characterized by markers of oxidative fibers (*Myh1*, *Myh2*, *Myl3*), neuromuscular junction genes (*Prkar1a*, *Chrne*), and oxidative metabolism (*Acadl*). Gene ontology analysis reinforced these findings as proximal–distal regions were enriched in processes such as glycolysis, skeletal muscle contraction, and glycogen metabolism, whereas central regions showed enrichment in fatty acid metabolism, the tricarboxylic acid (TCA) cycle, electron transport chain activity, and mitochondrial ATP production. Together, these results show a spatial organization of fiber type composition along the TA muscle, with glycolytic Myh4 myofibers enriched at the proximal/distal ends, and oxidative Myh1/Myh2 myofibers concentrated in the center. Using MALDI-MSI, the authors further mapped the spatial distribution of metabolites and lipids. Interestingly, they observed metabolic differences also between dorsal and ventral regions. To integrate these datasets, MSI data were binned to generate a pseudobulk mass spectrometry profile aligned with Tomo-seq sections. This integrated analysis highlighted a regional enrichment of glycolytic fibers in ventral and proximal–distal regions of the TA muscle, whereas oxidative fibers were predominantly localized dorsally and centrally.

Complementing these insights from mouse muscle, a proof-of-concept study in humans employed the Xenium platform to profile the transcriptomes of type I and type II fibers in skeletal muscle sections from young and old patients (Umek et al., 2025). The histological sections stained for MyHC isoforms were manually aligned with the Xenium output images. This approach enabled a separate analysis of the transcriptomic profiles in type I and type II muscle fibers (Umek et al., 2025). In type I fibers, the activated satellite cell marker LGR5 was more expressed, consistent with previous reports of higher satellite cell abundance in type I compared with type II fibers (Leung et al., 2020). Genes involved in lipolysis (*LPL*) and ketone body production (*HMGCS2*) were also enriched, in line with the oxidative metabolism characteristic of type I fibers. In contrast, type II fibers showed higher expression of *PVALB*, which encodes a Ca^2+^-binding buffer protein predominantly found in fast-twitch fibers (Schwaller, 2010). Age-related differences in extracellular matrix (ECM) and in structural components were also observed. In older muscle, genes such as *COL5A2* and *DES* were significantly more expressed. Collagens are critical for maintaining structural integrity, while desmin links myofibrils at the Z-discs, supporting fiber stability. These findings align with previous observations of increased expression of ECM and other structural components in aged muscle (Keele et al., 2023; Russ and Grandy, 2011). Direct comparisons between young and old patients further revealed fiber type–specific transcriptomic remodeling. In younger patients, both type I and type II fibers upregulate genes linked to contractile function, calcium handling, and metabolic efficiency, supporting higher regenerative and functional capacity (*ACTG2*, *S100A1*, *HMGCS2*). In contrast, older fibers show enrichment of genes involved in structural maintenance (*DES*, *DST*), stress responses (*MDM2*), and metabolic alterations (*PLIN4*, *GATM*), reflecting adaptations to age-related stress and degeneration.

Overall, this study highlights fiber type–specific transcriptomic profiles and the molecular changes associated with aging, demonstrating the power of spatial profiling to dissect both fiber-type differences and microenvironmental changes in human skeletal muscle.

Moving from age-related profiling to regenerative dynamics, Brorson et al. (2025) investigated skeletal muscle regeneration in elderly humans using spatial, temporal, and single-cell transcriptomics. Muscle injury was induced by electrically stimulated eccentric contractions of the vastus lateralis, with biopsies collected before and at 2, 8, and 30 dpi. Unsupervised clustering of the ST data identified 10 separate clusters: Type 1 and 2 muscle fibers (clusters 0–1), which decreased at 8 dpi but recovered by 30 dpi; clusters 3–5 reflected injury-related processes (cytoskeleton, proteasome, and erythrocyte functions) and were reduced at 8 dpi; cluster 9 (vascular/perivascular) remained stable. Dynamic clusters 6–8 were enriched in extracellular matrix genes (e.g., *COL1A2*, *POSTN*), immune response genes (e.g., *CD14*, *HLA-DRA*), and regenerative myofiber markers (e.g., *DES*, *MYH3*), respectively, all increased at 8 dpi with marked spatial confinement.

By integrating the ST data with previously published human skeletal muscle scRNA-seq (Farup et al., 2021), the authors were able to identify the contributions of individual cell types during regeneration. Early after injury (2 dpi), monocytes/macrophages, MuSCs, and endothelial cells peaked, indicating a rapid immune and stem cell activation. In contrast, FAPs, lymphocytes, and lymphatic endothelial cells reached their peak later, at 8 dpi, suggesting a sequential and coordinated cellular response. Colocalization analysis revealed that FAPs were closely associated with macrophages, MuSCs, and lymphocytes. Supporting this, ligand–receptor analysis uncovered extensive signaling from FAPs, particularly *via* Complement factor (C3)–ITGAX/ITGB2 and C3–ITGAM/ITGB2 interactions with macrophages. Follow-up *in vitro* experiments confirmed that human FAP-derived C3 recruits pro-inflammatory macrophages to injury sites, supporting phagocytosis and subsequent muscle regeneration.

Together, these studies demonstrate how ST provides new insights into the skeletal muscle biology. Regeneration-focused works (McKellar et al., 2023; McKellar et al., 2021; Brorson et al., 2025; Larouche et al., 2023) consistently reveal the dynamic interplay between MuSCs, FAPs, and immune cells showing how spatially confined cellular crosstalk and temporal coordination are essential for effective repair. Additionally, subsequent reanalysis (Young et al., 2022) revealed unexpected role for transient senescence in muscle repair. In parallel, studies on non-coding RNA biology (McKellar et al., 2023; Mcleod et al., 2024) demonstrate the unique ability of spatial methods to map the localization of lncRNAs and microRNAs, uncovering regulators of myogenesis and hypertrophy. Finally, fiber-type and metabolic profiling (D’Ercole et al., 2022; Martínez Mir et al., 2024; Umek et al., 2025) illustrates how ST can resolve the molecular architecture of oxidative and glycolytic regions, link metabolic pathways to fiber types, and uncover adaptations to aging and injury. Collectively, these works establish ST as a powerful tool for exploring fundamental mechanisms of muscle biology, especially during regeneration.

### 3.2 Muscle disorders

In parallel to studying healthy and regenerating muscle, multiple efforts have focused on leveraging ST to better understand the mechanisms underlying various muscular disorders, with particular emphasis on Duchenne Muscular Dystrophy (DMD). DMD is among the most severe pediatric degenerative myopathies. It is caused by mutations that result in the absence of functional dystrophin, a key protein responsible for stabilizing the muscle fiber membrane. The resulting membrane fragility leads to continuous cycles of muscle damage followed by regeneration (Duan et al., 2021). Different fiber types display differential sensitivity to damage, with type II fibers being preferentially affected compared to type I fibers (Webster et al., 1988). Muscle damage can occur in different fibers at different times, with each injury undergoing an independent repair process (Dadgar et al., 2014). As a result, lesions at different stages of regeneration coexist within the muscle. Indeed, early work from Dadgar et al. (2014), using laser capture microdissection, identified patterns consistent with asynchronous remodeling in DMD muscle and demonstrated that regions located in between asynchronous regenerative areas display aberrant fibrotic infiltration (Dadgar et al., 2014) This indicates that one of the contributing factors to DMD pathogenesis is the focal and uncoordinated response to injury. ST approaches have been also applied to other muscle-related conditions, for example, to Amyotrophic Lateral Sclerosis (ALS), a progressive neurodegenerative disease characterized by the vulnerability of both NMJs and muscles, particularly fast-twitch fibers (Ruggieri et al., 2025). Similarly, heterotopic ossification (HO), the abnormal formation of bone within muscle tissue triggered by trauma or genetic factors, has also been investigated using ST (Kan et al., 2024).

Heezen et al. (2023) applied Visium ST to investigate DMD pathology in muscle tissues from two dystrophic mouse models, mdx and D2-mdx, alongside their respective controls (C57BL10 and DBA/2J) at 10 weeks of age (Heezen et al., 2023). Despite a limited sample size, their proof-of-concept study effectively captured the hallmark histological features of dystrophic tissue. Notably, they identified distinct pathological clusters, including regenerating fibers, regenerating fibers with localized inflammation, necrotic fibers infiltrated by macrophages, and regions containing adipocytes. In the more severely affected D2-mdx model, they also observed clusteres characterized by inflammation and calcification, as well as necrotic fibers. To identify genes associated with muscle regeneration, they compared regions of active regeneration with intact muscle regions within the same sample. The analysis revealed increased expression of *Myl4*, *Sparc*, and *Hspg2* in regenerating regions from mdx, whereas in D2-mdx mice, *Vim*, *Fn1*, and *Thbs4* were upregulated in fibrotic areas, while *Bgn*, *Ctsk*, and *Spp1* were enriched in calcified regions. Next, to test if the histopathological changes observed in dystrophic muscle were associated with regional transcriptional reprogramming, the authors performed RNA velocity analysis on the D2-mdx. Among the top drivers of the transition, they identified Galectin-3 *(Gal-3)*, mainly expressed in macrophages, highlighting the role of immune cells in DMD progression.

As multiple lines of evidence indicate, macrophage activity is deregulated in DMD (Dadgar et al., 2014; Villalta et al., 2009; Mann et al., 2011). To investigate how macrophages contribute to fibrosis development during disease progression, Coulis and colleagues combined scRNAseq and ST (Coulis et al., 2023). scRNA-seq was first used to characterize macrophages from healthy and dystrophic muscle, identifying six distinct clusters that did not reflect the traditional M1 or M2 macrophage classifications. In dystrophic muscle, the predominant macrophage population was Gal-3^+^ macrophages, which expressed several profibrotic factors, including *Spp1*, as previously reported by Heezen (Heezen et al., 2023). The authors then performed ST on the gastrocnemius/plantaris muscle of 6-week-old D2-mdx mice and identified areas with high *Gal-3* expression overlapping with degenerative lesions. Differential gene expression analysis between *Gal-3^+^* and *Gal-3^-^* -areas revealed high expression of fibrosis related genes (*Mmp12*, *Fn1*, *Postn*, *Tgfβ*, *Pdgfr*). Further immunofluorescence analysis confirmed PDGFRα^+^ stromal cells in the degenerative regions alongside with Gal-3^+^ macrophages. SnRNA-seq reference data and *in vitro* analysis further suggested that *Gal-3+* macrophages may interact with FAPs in dystrophic muscle *via* *Spp1* pathway both in mouse and DMD patient samples. These results identify *Spp1* as a potential regulator of *Gal-3^+^* macrophage and FAP interaction.

In a complementary approach, Patsalos et al. (2024) generated an immune-specific single-cell and ST datasets of regenerating muscle after acute injury and during early dystrophy, providing detailed spatial annotation of myeloid subpopulations with enhanced subspot resolution (Patsalos et al., 2024). They performed parallel CD45^+^ scRNA-seq and ST on the gastrocnemius muscle of 2-month-old D2-mdx mice. Their analysis identified several distinct subsets of monocytes, macrophages (MFs), and dendritic cells (DCs) and identified a set of colocalizing cell types during the regenerative inflammation phase.

Glucocorticoids (GC) are commonly used to treat DMD to delay disease progression, although long-term daily use is limited by serious side effects (e.g., bone demineralization and muscle atrophy) (Quattrocelli et al., 2017). To explore the response of the immune cells to therapeutic modulation, the authors next investigated the effects of intermittent GC treatment in young D2-mdx mice. In untreated dystrophic muscle, they identified distinct regenerative inflammation zones (RIZs) with a layered structure: proinflammatory macrophages surrounding necrotic fibers, resolution-related macrophages forming a barrier, and regenerating muscle fibers at the periphery. Prednisolone treatment disrupted this organization, notably eliminating the regenerating outer layer and causing an overlap between inflammatory and resolving macrophage layers. This disorganization could potentially impact the ability to regenerate extensive lesions. Additionally, they observed increased spatial expression of GC receptor targets and atrophy-associated genes alongside reduced expression of inflammatory, ECM, and regenerative genes. The identification of distinct damage-clearing RIZs in early dystrophic muscle offers a valuable means to monitor disease progression and evaluate therapeutic responses. Moreover, it may guide the development of strategies to preserve regenerative niches in DMD and potentially other chronic inflammatory diseases.

Recently, Jeon et al. (2025) investigated the pathogenic features of DMD and BMD Becker Muscular Dystrophy, as well as the palliative effects of glucocorticoid treatment, using an integrated snRNA-seq and ST approach (Jeon et al., 2025). They performed snRNA-seq on both DMD and BMD patient samples and conducted ST profiling on matched muscle biopsies from DMD patients only. Using the Cell2Location, they identified spatial co-localization patterns between satellite cells, myeloid cells, and lymphoid cells within the dystrophic muscle microenvironment. Furthermore, comparative analysis between DMD and BMD samples revealed elevated *EZH2* expression in proliferating satellite cells. Pharmacological inhibition of EZH2 promoted muscle regeneration by enhancing myogenic differentiation. *In vivo*, D2-mdx mice were treated with EZH2 inhibitors (GSK126 and tazemetostat), either alone or in combination with the glucocorticoid deflazacort. Their results demonstrated that EZH2 inhibition not only improved the dystrophic muscle phenotype but also attenuated the adverse effects typically associated with deflazacort therapy.

Additional insights into the inflammatory process in DMD were provided by the work of Stec et al. (2023), which analyzed muscles from 6-week old WT and D2-mdx mice using ST (Visium). Their analysis focused on clusters present in the D2-mdx dataset, including two distinct immune clusters, one encompassing regenerating myofibers, and a hybrid one expressing both myofiber and immune-related genes (Stec et al., 2023). The first immune cluster (Immune 1) was enriched for genes linked to granulocyte/neutrophil migration, along with myofiber contractile proteins, suggesting active damage and early regeneration. The second Immune cluster (Immune 2) showed the presence of antigen-presenting cell (APC) and extracellular matrix genes, pointing to a more intermediate regenerative phase. Histological analysis supported these findings showing that the first immune cluster areas contained damaged myofibers with invading cells and proinflammatory macrophages, while the other had fewer myofibers and abundant macrophages, but lacked anti-inflammatory macrophages. In contrast, regenerating fiber clusters showed new myofibers surrounded by fewer immune cells. In undamaged type IIB myofiber regions of D2-mdx mice, the authors observed upregulation of immune and fibrotic gene pathways compared to healthy muscles. Furthermore, neighborhood enrichment analysis revealed a spatial association between Immune regenerative cluster and clusters enriched for mesenchymal stromal progenitor cells. In contrast, the more inflammatory Immune clusters were found adjacent to fiber clusters expressing inflammation-associated genes. This spatial expansion of fibrotic and immune signals propagates beyond the primary lesion, contributing to the amplification of pathology throughout the muscle.

While multiple studies focused on immune and fibrotic processes in DMD, ST approaches have been also applied to other muscle-related conditions. For example, following up our initial work on denervation we investigated how altered innervation interferes with muscle homeostasis in ALS, (Amyotrophic Lateral Sclerosis) a progressive neurodegenerative disease characterized by the vulnerability of both NMJs and muscles, particularly fast-twitch fibers (D’Ercole et al., 2022). To investigate the spatial dynamics of muscle degeneration during disease progression, we analyzed TA muscle from the SOD1^G93A^ ALS mouse model at early and late disease stages using the Visium platform (Ruggieri et al., 2025). Similarly to what we found in our previous work we identified 10 major clusters defined by canonical markers. Among these, glycolytic type IIB myofibers—known to be especially susceptible to disrupted innervation in ALS, exhibited pronounced transcriptional changes. In contrast type IIA-IIX fibers displayed an increased energy demand but also signs of impaired mitochondrial functions. Notably, the enhanced atrophy observed in type IIB myofibers was associated with dysregulation of the polyamine pathway. Restoring polyamine homeostasis in ALS mice rescued the muscle phenotype, highlighting the therapeutic potential of targeting this pathway.

Similarly, heterotopic ossification (HO), the abnormal formation of bone within muscle tissue triggered by trauma or genetic factors (Dey et al., 2017), has also been investigated using combined scRNA-seq and ST methods. Kan and colleagues analyzed uninjured and injured tibial muscles at 1, 3 and 7 dpi from the Nse-Bmp4 mice (neuron specific enolase, bone morphogenetic protein 4), a genetic model of HO (Kan et al., 2024). As hyperactive inflammation is a well-known driver of abnormal tissue repair during HO, the authors aimed to investigate the interactions between immune cells and mesenchymal stem cells (MSCs). Clustering the ST data identified distinct regions in the injured muscle enriched for both MSC and immune cell markers. Cell-to-cell interaction analysis showed that the immune microenvironment, especially macrophage subtypes (M1 and M2), regulates MSCs behavior. M1 macrophages promote MSCs proliferation, while M2 macrophages support differentiation. Indeed, quiescent MSCs were mainly localized in uninjured tissue, while cycling MSCs accumulated within lesions at 1 and 3 dpi, followed by differentiating MSCs at later stages. Monocytes/macrophages were found to co-localize with cycling and differentiating MSCs. Lastly, the authors identified STAT signaling, CD44, and OSM-OSMR pathways as key molecular regulators of MSC transition. These findings shed light on how the immune microenvironment drives MSCs transitions during HO, highlighting potential therapeutic targets to control aberrant bone formation.

Together, these studies demonstrate how ST has changed our understanding of muscle pathology. Heezen et al. (2023) demonstrated the ability of ST to resolve hallmark histopathological features in mdx and D2-mdx mice, identifying regenerating, necrotic, fibrotic, and calcified regions, and implicating Galectin-3–expressing macrophages in disease progression. Coulis extended these findings by combining scRNA-seq and ST to show that Gal-3+ macrophages interact with FAPs *via* Spp1, driving fibrosis (Coulis et al., 2023). The work from Patsalos (Patsalos et al., 2024) generated immune-focused scRNA-seq and ST datasets, defining distinct macrophage and dendritic cell subsets within regenerative inflammation zones and showing how glucocorticoids disrupt their organization. Jeon and collaborators (Jeon et al., 2025) integrated snRNA-seq and ST in human DMD and BMD samples, identifying spatial proximity between satellite and immune cells, and highlighting EZH2 as a therapeutic target. Complementing these immune-focused studies, Stec mapped immune and stromal interactions in D2-mdx muscle, showing how fibrotic signals expand beyond lesions and propagate pathology (Stec et al., 2023). In addition to DMD, applications in ALS and heterotopic ossification highlight the broader utility of ST in uncovering fiber type–specific vulnerabilities and immune–mesenchymal crosstalk during aberrant tissue remodeling. However, the potential applications of ST can extend beyond regeneration and degeneration processes. As discussed, type I (oxidative) and type II (glycolytic) fibers differ in their metabolic properties, which influences their susceptibility to different pathological features. For example, in metabolic diseases, type I fibers are more prone to intracellular lipid accumulation (Umek et al., 2021). ST enables studying the correlation of fiber type identity with local pathological changes, including fibrosis, inflammatory cell infiltration or lipid accumulation. Applying ST could therefore help to uncover how specific fiber types contribute to disease progression. By capturing the spatial organization of cellular niches, ST not only refines our view of disease mechanisms but also provides a framework for evaluating therapies and identifying new intervention points across muscular disorders.

## 4 Current limitations and future perspectives

ST technologies face several limitations that impact their efficiency and applicability, particularly in relation to transcript capture efficiency, sample quality, and preparation. These technical constraints can significantly influence both the resolution and interpretability of ST data. A primary challenge is RNA capture efficiency. While *in situ* imaging-based approaches can localize hundreds to thousands of genes, they do not provide full transcriptome coverage. Array-based techniques offer the potential for comprehensive sequencing, but capture efficiency remains a constraint, limiting transcript detection. This issue affects the depth and coverage compared to bulk transcriptomics, making it difficult to identify low-abundance transcripts. Furthermore, implementing ST is considerably more expensive than traditional bulk RNA sequencing. The increased costs are attributed to the need for specialized equipment and reagents, the complexity of sample preparation, and the extensive data analysis required. These factors collectively make ST more costly than conventional transcriptomic methods, thus limiting broad access to this technology (Vandereyken et al., 2023). Another critical limitation regarding transcript coverage is that several ST techniques rely on probes to capture or hybridize RNA transcripts. As a result, the data may show biases in the representation of certain RNA species, as their accurate detection relies on probe efficiency (Du et al., 2023).

As with many other techniques, an additional challenge lies in the collection and preservation of tissue samples, especially with human biopsies. This issue is particularly evident when collecting samples from healthy individuals, which are typically less accessible than those from individuals with pathological conditions. It is also challenging to collect samples from the same muscle type, or ideally the same anatomical region. The collection site is critical, as muscle function, workload and anatomical location influence fiber metabolism, which in turn affects gene expression, activation of cellular pathways, and overall responses to disease. Another major challenge is assembling a sufficiently homogeneous cohort of patients with similar age, sex, or pathological history. The complexity of sample preparation further adds to these difficulties. The process includes multiple steps, from tissue harvesting to cryosectioning, depending on the specific requirements of the chosen method. For muscle tissue, in particular, the freezing and storage steps are critical to ensure high tissue quality and, consequently, to produce accurate spatial maps during the analysis phase. FFPE tissues generally yield lower-quality RNA and DNA compared to FF samples (Yi et al., 2020). Formalin fixation induces nucleic acid fragmentation, protein cross-linking, and chemical modifications, compromising their integrity and suitability for downstream applications such as PCR and sequencing. In contrast, fresh frozen tissues better preserve nucleic acids by preventing enzymatic degradation, making them the preferred choice for molecular analyses where high-quality RNA and DNA are required (Steiert et al., 2023).

Furthermore, even after a successful experimental run, additional issues may arise during the analysis as the overall size of data generated demands substantial computational power and storage. Moreover, the analytical workflow demands both a deep understanding of cellular and tissue biology and the application of sophisticated statistical models. As the field is still relatively young, standardized protocols and benchmarks are under active development. Consequently, strong collaboration between computational and experimental researchers is essential, an approach that can be challenging to implement in laboratories lacking dedicated bioinformatics expertise. Despite these challenges, ongoing advancements in ST are focused on improving sensitivity, spatial resolution, and cost-effectiveness, broadening its applicability in research and clinical diagnostics.

Future techniques should aim to integrate ST with complementary omics technologies, such as proteomics, epigenomics, and metabolomics, to provide a more comprehensive view of cellular function. For example, as discussed in Section 3.1 Healthy and regenerating muscle, Martínez Mir et al. (2024) combined two spatial omics approaches: RNA tomography (Tomo-seq) and matrix-assisted laser desorption/ionization mass spectrometry imaging (MALDI-MSI). MALDI-MSI enables spatial detection of metabolites and lipids, while Tomo-seq was used to identify fiber types and perform differential gene expression analysis. To integrate the datasets, the MALDI-MSI data was binned to generate a pseudobulk metabolomics profile along the proximal–distal axis, parallel to the Tomo-seq sections. This alignment allowed metabolites from MALDI-MSI to be directly compared with gene expression profiles from Tomo-seq, enabling correlation analyses that link transcriptomics with local metabolic states of myofibers.

A similar multiomics approach could be applied to study muscle degeneration by integrating ST with proteomics. To date, this has not been extensively explored in the field of neuromuscular diseases or muscle regeneration. However, Gouin and colleagues demonstrated the potential of such approach by combining ST with spatial proteomics to analyze tissue samples from patients with high-grade muscle-invasive bladder cancer (Gouin et al., 2021). In their study, ST was the primary analytical focus, while spatial proteomic data were used to validate transcriptomic findings. To further confirm cell co-localization at single-cell resolution, they performed a 35-plex immunohistochemistry panel using the Co-detection by Indexing (CODEX) platform on tumor tissue microarrays from the same patient cohort. Importantly, proteomics provides an additional validation to ST, since transcriptomic data alone cannot determine which genes are ultimately translated into proteins and thus functionally active.

## 5 Conclusion

The integration of ST into skeletal muscle research marks an important advancement in our ability to study tissue architecture alongside gene expression. As the field evolves, innovations in resolution, sensitivity, protocols, and commercial platforms are reshaping our understanding of tissue biology - particularly in skeletal muscle, where spatial context is essential to characterize regeneration, fiber-type distribution, inflammatory microenvironments, and their contributions to disease mechanisms. Recent studies applying ST to DMD—and more broadly in the entire muscle field—clearly illustrate this potential. By leveraging ST and single-cell technologies, researchers have identified gene expression patterns and cell populations that drive regeneration, mediate inflammatory and fibrotic signaling from damaged muscle areas, or contribute directly to disease progression. Ultimately, these findings highlight how surveying gene expression within specific microenvironments can reveal novel therapeutic pathways, emphasizing the potential impact of spatially resolved transcriptomics in translational research.

Despite these advances, important challenges remain. The lack of standardized protocols and benchmarks complicates cross-study comparisons and limits reproducibility, emphasizing the need to harmonize analytical workflows as ST moves toward clinical applications. New computational strategies, including AI (Artificial Intelligence) and multiomic integration, can address the analytical demands of increasingly large and complex datasets. Additionally, constructing spatial reference atlases of healthy tissues will provide critical baselines for mapping disease-associated changes. As ST platforms continue to improve in accessibility and cost, the technology promises to transform not only our understanding of muscle biology, but also to pave the way for faster, more accurate diagnosis and novel therapeuticS strategies.
